# Electrochemical Aptamer-Based Biosensors for Sepsis Diagnosis: Recent Advances, Challenges, and Future Perspectives (2020–2025)

**DOI:** 10.3390/bios15070402

**Published:** 2025-06-20

**Authors:** Ling Ling Tan, Nur Syamimi Mohamad

**Affiliations:** Southeast Asia Disaster Prevention Research Initiative (SEADPRI), Institute for Environment and Development (LESTARI), Universiti Kebangsaan Malaysia, UKM Bangi, Bangi 43600, Selangor, Malaysia; lingling@ukm.edu.my

**Keywords:** aptamer, biomarker, biosensor, electrochemistry, nanotechnology, sepsis

## Abstract

Sepsis remains a global health emergency, demanding timely and accurate diagnostics to reduce morbidity and mortality. This review critically assesses the recent progress (2020–2025) in the development of electrochemical aptamer-based biosensors for sepsis detection. These biosensors combine aptamers’ high specificity and modifiability with the sensitivity and miniaturization potential of electrochemical platforms. The analysis highlights notable advances in detecting key sepsis biomarkers, such as C-reactive protein (CRP), procalcitonin (PCT), interleukins (e.g., interleukin-6 (IL-6), tumor necrosis factor-alpha (TNF-α)), lipopolysaccharides (LPSs), and microRNAs using diverse sensor configurations, including a field-effect transistor (FET), impedance spectroscopy, voltammetry, and hybrid nanomaterial-based systems. A comparative evaluation reveals promising analytical performance in terms of the limit of detection (LOD), rapid response time, and point-of-care (POC) potential. However, critical limitations remain, including variability in validation protocols, limited testing in real clinical matrices, and challenges in achieving multiplexed detection. This review underscores translational barriers and recommends future directions focused on clinical validation, integration with portable diagnostics, and interdisciplinary collaboration. By consolidating current developments and gaps, this work provides a foundation for guiding next-generation biosensor innovations aimed at effective sepsis diagnosis and monitoring.

## 1. Introduction

Sepsis, a life-threatening organ failure caused by a dysregulated host response to infection, is a global health crisis, impacting an estimated 48.9 million people each year and accounting for 11 million fatalities globally [1]. This septic infection has a significant negative impact on healthcare systems in terms of both public health and economics, especially in low- and middle-income countries (LMICs) where fatality rates might surpass 40% [2,3]. Therefore, prompt and precise diagnosis is essential for lowering mortality. However, the clinical signs of sepsis are often ambiguous, which causes delays in diagnosis and treatment. Due to patient individuality and pathogen diversity, conventional diagnostic techniques, such as blood cultures (BCs) and biochemical assays, are not only time-consuming but also prone to false-negative results [4,5]. These drawbacks highlight the critical need for quick, accurate, and sensitive diagnostic strategies that can support early detection and treatment.

Therefore, a biosensor is an effective alternative, since it offers speed, specificity, and the possibility of real-time monitoring [6,7,8,9,10]. Significant progress has been made in biosensor technology in recent years to identify sepsis biomarkers, such as procalcitonin (PCT), C-reactive protein (CRP), and interleukins (ILs), each of which provides important information on inflammation and early immunological responses [11,12,13]. The specificity and adaptability of optical sensors, especially those that employ aptamer-based techniques, are excellent for detecting biomarkers, such as CRP and interleukin-6 (IL-6) [14,15]. To achieve great sensitivity and facilitate early sepsis identification, these sensors make use of gold nanorods based on localized surface plasmon resonance (LSPR) and a graphene oxide/nickel/platinum nanoparticle micromotor (MM) with fluorescence detection, respectively [14,15].

Other than this, electrochemical sensors are being emphasized for their affordability and versatility in point-of-care (POC) diagnostics, which makes them a viable choice for sepsis because of their quick reaction time and excellent sensitivity, even at low concentrations of biomarkers [16,17,18]. More precisely, inventive possibilities for improving sensor performance have been made possible by the incorporation of nanomaterials and aptamer-based recognition components into biosensor technology [19,20,21]. While aptamers, a single-stranded DNA or RNA molecule with a high binding affinity, offer an excellent replacement for antibodies in biomarker recognition, particularly for unstable proteins in complex biological samples, nanomaterials like gold nanoparticles (AuNPs), carbon nanomaterials, and quantum dots have shown exceptional electrical and optical properties [22,23,24,25,26,27].

Despite these developments, there is still a significant gap between clinical adoption and the advancement of technology, as only a small portion of biosensor-based diagnoses have made it to clinical practice. This is due to challenges involving regulatory approval, protocol uniformity, scalability, and reproducibility across various patient populations. This disparity highlights the need for thorough validation studies that improve biosensor sensitivity and specificity while also guaranteeing dependability in real-world settings. This is particularly important in areas where sepsis is endemic and resources for sophisticated diagnostics are limited [28,29,30].

Considering these challenges, this review provides a comprehensive analysis of the recent advancements in biosensor technology for sepsis diagnosis, with an emphasis on various approaches for biomarker detection, signal augmentation, and device miniaturization for POC applications. By assessing the efficacy of various sensor types and the role of aptamers and nanomaterials, the practical challenges to clinical implementation are highlighted. This paper also aims to present a thorough analysis of the state of sepsis biosensors based on aptamer and electrochemical transduction, highlighting key advancements as well as areas still in need of further research.

## 2. Clinical Overview of Sepsis and Diagnostic Methods

### 2.1. Definition and Progression of Sepsis Clinically

**Figure 1:**
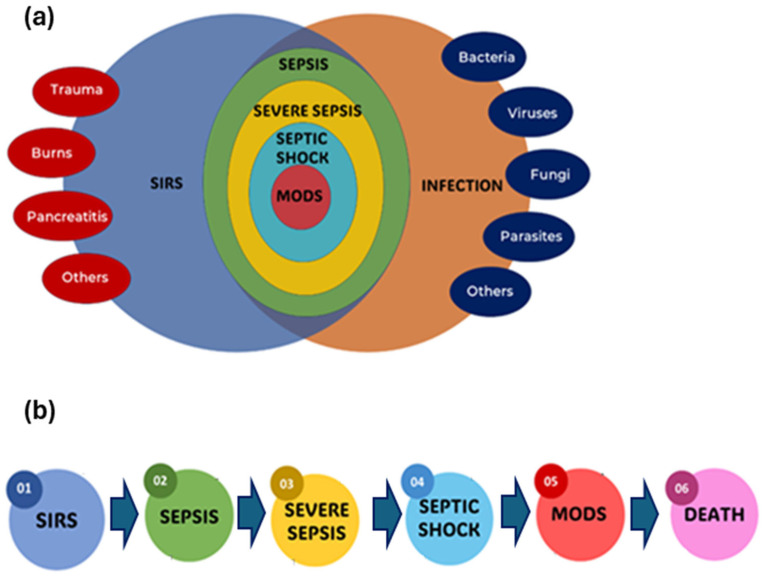
A conceptual overview and diagnosis of systemic inflammatory response syndrome (SIRS), infection, sepsis, severe sepsis, septic shock, multiple organ dysfunction syndrome (MODS), and death. (**a**) Complex interrelationships; (**b**) progression from SIRS to death.

In 1991, the American College of Chest Physicians and the Society of Critical Care Medicine (ACCP/SCCM) conducted a conference to develop more specific criteria for sepsis and related disorders [31]. They presented broad categories for sepsis and systemic inflammatory response syndrome (SIRS), as well as comprehensive physiological indicators for patient categorization. They introduced new terminology while eliminating others. Severe sepsis, septic shock, hypotension, and multiple organ dysfunction syndrome (MODS) were all described using precise criteria. Adapted from Bone et al. [31], Figure 1 provides a comprehensive overview of the interrelationship and clinical progression of SIRS, infection, sepsis, severe sepsis, septic shock, and MODS, all of which can culminate in fatal outcomes if not managed effectively.

This figure depicts a bidirectional relationship between infection and sepsis. Infection with pathogens, such as bacteria, viruses, fungi, or parasites, causes an immune response to eliminate the invading germs. However, sepsis can result from an excessive or uncontrolled immunological response. Sepsis, on the other hand, can prolong infection by suppressing the immune system, reducing the body’s ability to fight the pathogen. Beyond severe sepsis, the most crucial stage is septic shock, as seen in Figure 1. Septic shock is distinguished by severe circulatory and cellular/metabolic abnormalities, resulting in abrupt circulatory collapse. Despite appropriate fluid resuscitation, patients in septic shock experience chronic hypotension, defined as a prolonged period of mean arterial pressure (MAP) below 65 mmHg, and often require vasopressor therapy to maintain adequate tissue perfusion. This development emphasizes the severity of the inflammatory response and its potential to result in life-threatening diseases. This bidirectional link highlights the complex nature of sepsis and the significance of prompt management to prevent it from progressing to severe sepsis, septic shock, and MODS, stressing the condition’s growing severity.

Figure 1a illustrates the complex interrelationships between SIRS, sepsis, severe sepsis, septic shock, and infection. At the heart of this image is SIRS, which reflects the body’s general response to numerous insults, such as infection, trauma, and inflammation. When an infectious pathogen causes SIRS, it might serve as a prelude to more serious illnesses. The Venn diagram depicts the path from SIRS to sepsis, which happens when the body’s reaction to infection becomes dysregulated, resulting in a systemic inflammatory condition. This dysregulated response can result in life-threatening consequences, necessitating early detection and care. Furthermore, the graphics depict the progression to severe sepsis, which is distinguished by the appearance of organ dysfunction because of the strong inflammatory response. Organ dysfunction can show in a variety of ways, including altered mental status, decreased urine output, respiratory failure, or hypotension, dramatically increasing the risk of death (Figure 1b).

Figure 1b depicts a detailed representation of the sequential stages of sepsis, demonstrating that the progression of the medical condition depends on the time of diagnosis and the action taken. Sepsis frequently evolves in chronological order from SIRS, to severe sepsis, and finally, to septic shock. SIRS is defined as the presence of two or more of the following key physiological markers: an abnormal body temperature (greater than 38.0 °C or less than 36.0 °C), a high heart rate (higher than 90 beats min^−1^), an increased respiratory rate (higher than 20 breaths min^−1^ or arterial carbon dioxide tension below 32 mm Hg), or an abnormal white blood cell count (higher than 12,000 µL^−1^, lower than 4000 µL^−1^, or including more than 10% bands). Sepsis is diagnosed when SIRS criteria are met in the presence of a proven or suspected infection. Meanwhile, MODS is the final stage of severe SIRS, or sepsis. MODS is defined as a clinical syndrome characterized by the onset of progressive and potentially reversible physiological dysfunction in two or more organs or organ systems caused by a range of acute insults, including sepsis.

However, the outcome, including the development of MODS and mortality, is unpredictable and depends on a variety of factors, such as the timing and efficacy of therapy treatments. Thus, Figure 1, which depicts the sequential progression from SIRS to sepsis, severe sepsis, and septic shock, provides a comprehensive overview of the systemic inflammatory response and its relationship to infection, assisting clinicians in understanding and managing these complex conditions. Hence, early detection and adequate therapeutic interventions can change this trajectory, potentially averting the progression and highly influencing the clinical course of sepsis.

### 2.2. Diagnostic Techniques for Sepsis: Conventional and Advanced Analytical Approaches

Sepsis/septic shock is a potentially fatal and time-sensitive illness that requires prompt treatment to avoid death at some point. The diagnostic landscape of sepsis includes conventional methods, which are widely used in clinical settings, and advanced analytical techniques, which aim to improve sensitivity, specificity, and speed of detection. Various conventional diagnostic methods have been used to detect sepsis-related pathogens, including culture-based approaches, most notably, blood cultures (BCs), which remain the gold standard for identifying and isolating infectious agents from sterile body fluids [32]. The utilization of polymerase chain reaction (PCR)-based techniques has significantly advanced the diagnosis of sepsis. PCR possesses the capability to amplify bacterial DNA faster than BCs, resulting in more rapid findings [33]. In other words, PCR techniques can be used in detecting low amounts of target DNA. Next, the state of the art in sophisticated diagnostic instruments is represented by mass hybridization spectroscopy and microarray technology [34,35]. Despite its quick multiplex analytical capabilities, mass hybridization spectroscopy, like MALDI-TOF, is limited by its expensive equipment, lengthy sample preparation requirements, and sensitivity to matrix effects. High-throughput screening is possible with DNA microarray technologies, but they have drawbacks, like cross-hybridization artifacts, low probe specificity, and complicated data processing needs.

However, despite their dependability, these approaches have certain drawbacks. The necessity for innovation in sepsis diagnosis cannot be overstated. Current diagnostic methods, while effective, suffer from time lags and limitations in pathogen detection. Advancements such as nanotechnology-based biosensors, artificial intelligence, and machine learning models are being explored to enhance diagnostic accuracy, speed, and adaptability. These emerging technologies could revolutionize sepsis diagnostics, making it possible to detect the condition earlier and more accurately in order to guide targeted therapy, ultimately improving patient outcomes and reducing mortality.

#### 2.2.1. Blood Cultures (BCs)

Blood cultures (BCs), which are frequently considered the gold standard for detecting bloodstream infections, continue to provide the foundation for the diagnosis of sepsis [36,37]. Blood samples are incubated during the procedure to promote the growth of infections, which are then detected using molecular or biochemical techniques. Even though these techniques are frequently employed, they are usually slow, requiring 24 to 72 h for conclusive results [38]. This might cause patients to miss out on important medical interventions, like the prescription of the right medications for patients. Also, this delay can be life-threatening in cases of severe sepsis, where early intervention is critical to patient survival. A false positive occurs commonly due to skin contamination (e.g., coagulase-negative staphylococci). False negatives may also occur due to prior antibiotic treatment or particularly for pathogens that require highly specific and enriched media for optimal growth, such as certain anaerobic or slow-growing bacteria, leading to diagnostic inaccuracies. Rapidly developing sepsis puts temporal constraints on treatment options, emphasizing the necessity for quicker diagnostic turnaround times.

Additionally, blood cultures have limited sensitivity, especially when the pathogen load is low, as is often the case in patients with severe infections or those who have been partially treated with antibiotics [39]. Another limitation is the difficulty in detecting fungal and viral causes of sepsis, which further constrains its utility as a universal diagnostic tool [40]. The technique’s dependence on large blood volumes, which may be problematic for pediatric or critical patients, further complicates its routine use [41]. Therefore, while blood cultures provide a foundation for identifying pathogens, this method is far from perfect for time-sensitive, complex conditions like sepsis. Despite these challenges, blood cultures remain indispensable in clinical settings, especially for antibiotic susceptibility testing [42]. They offer valuable information about antimicrobial resistance patterns, which is crucial for tailoring patient-specific treatment regimens. This utility, combined with its low cost and accessibility, ensures its continued relevance in sepsis diagnostics. However, the slow nature of blood cultures necessitates the incorporation of complementary techniques to improve diagnostic efficiency.

#### 2.2.2. PCR-Based Techniques

Sepsis diagnostics have advanced significantly with the use of polymerase chain reaction (PCR)-based methods [43,44]. In contrast to the days needed for conventional blood cultures, PCR allows for the detection of pathogens in a matter of hours by amplifying specific DNA or RNA sequences [45,46]. This speed can have a significant impact on sepsis management, since prompt detection enables adequate and timely antimicrobial therapy. The capacity of real-time PCR to measure the bacterial load and evaluate the course of illness has led to its widespread adoption [47,48]. Despite its widespread use, the PCR methodology has several drawbacks, including the need for specialized primers, which limit detection to known infections with well-established genetic markers, and sample contamination [49]. Particularly, it can compromise diagnostic accuracy, leading to potentially serious consequences for patients, as highlighted in several false-positive cases reviewed by Borst and colleagues [50].

False positives due to contamination and false negatives due to a primer mismatch or low pathogen concentration also complicate the diagnostic process. Moreover, PCR does not typically provide information on antimicrobial resistance, which is essential for guiding appropriate therapy. Another consideration is the cost and complexity of PCR-based techniques. These methods require specialized equipment and expertise, making them less accessible in resource-limited settings. Additionally, PCR’s sensitivity may be compromised by the presence of host DNA or other interfering substances in blood samples, further complicating its application in clinical practice [51]. While multiplex PCR systems that target multiple pathogens simultaneously have been developed, they still face challenges in balancing sensitivity with specificity.

Despite these limitations, PCR has proven invaluable in reducing time to diagnosis in sepsis cases. Its integration with other molecular techniques, such as next-generation sequencing (NGS), holds promise for improving its sensitivity and scope [52]. In combination with rapid phenotypic methods, PCR could serve as a complementary tool that addresses the shortcomings of both molecular and conventional diagnostic methods.

#### 2.2.3. Spectroscopy-Based Approaches

By evaluating mass spectrometry data, spectroscopy provides excellent sensitivity in identifying pathogen-specific biomarkers, enabling accurate identification of bacterial proteins and metabolites [35]. Conversely, microarray technology allows for the hybridization of numerous DNA probes to enable the simultaneous identification of a wide variety of pathogens [34]. Both methods have the potential to deliver quick, precise findings, but they still need to be improved before they can be completely incorporated into standard clinical practice. Spectroscopy, particularly through techniques like Matrix-Assisted Laser Desorption/Ionization Time-of-Flight (MALDI-TOF) mass spectrometry, has revolutionized the rapid identification of pathogens in sepsis [53]. MALDI-TOF analyzes the unique protein profiles of microbial organisms, enabling the identification of bacteria, fungi, and other pathogens in minutes once they are cultured. This method’s precision and speed have made it an essential complement to conventional blood cultures, drastically reducing the time required for pathogen identification from days to hours. Its ability to identify a wide range of organisms with high accuracy has elevated it to a pivotal role in advanced diagnostic laboratories.

Mass spectrometry’s benefits, however, are contingent on pathogen culture, meaning it shares the limitations of BC techniques, such as false negatives in patients who have received prior antibiotic treatment. Despite its high-throughput capabilities, MALDI-TOF is constrained by its inability to detect antimicrobial resistance, necessitating additional phenotypic or genotypic testing to determine appropriate treatment regimens. There is also a need for well-maintained microbial databases to ensure the accuracy of pathogen identification, which can be a limitation in under-resourced settings. Recent developments in mass spectrometry have focused on direct-from-blood applications, bypassing the need for cultures and offering even faster diagnostics. While promising, these technologies are still in the developmental phase and face hurdles, such as high costs and the requirement for highly trained personnel. Moreover, as these methods evolve, there is an increasing demand for standardized protocols and database harmonization to ensure that results are comparable across different settings.

Spectroscopy via hybridization techniques, including surface plasmon resonance (SPR) and Raman spectroscopy, is also being explored for real-time, label-free detection of sepsis-related biomarkers. These methods offer high sensitivity and the ability to monitor biomolecular interactions in real time without labels or complex sample preparation. For example, Hosseinniay and co-workers [14] developed an SPR-based optical nanobiosensor using gold nanorods functionalized with CRP-specific aptamers for the detection of C-reactive protein. Their system achieved a linear detection range between 2–20 nM with a limit of detection (LOD) of 2 nM and showed strong selectivity against TNF-α and BSA, confirming its diagnostic relevance in inflammatory conditions, such as sepsis. These approaches, though still experimental, offer the possibility of rapid and highly sensitive detection of sepsis-related biomarkers. As these technologies mature, they may offer the potential to outperform traditional culture-dependent methods and PCR-based approaches.

#### 2.2.4. Nanotechnology-Based Sensors/Biosensors

Nanotechnology-based sensors and biosensors have recently gained significant attention as powerful tools for the early detection and management of sepsis. These innovative platforms take advantage of the unique properties of nanomaterials, such as gold nanoparticles (AuNPs), graphene, and quantum dots. Thus, by enhancing sensitivity and specificity and enabling real-time detection, they hold promise for point-of-care (POC) diagnostics, where quick decision-making can be the difference between life and death. For instance, portable biosensors equipped with these nanomaterials can deliver near-instant results, allowing healthcare providers to initiate timely and aggressive interventions. Among their many applications, nanomaterials serve as signal amplifiers that help detect critical sepsis biomarkers, like procalcitonin (PCT) and C-reactive protein (CRP), at very low concentrations. Papafilippou et al. [54] concentrated on optical and magnetic amplification strategies using AuNPs, quantum dots, and magnetic nanoparticles, presenting them as versatile tools for both pathogen detection and biomarker quantification. These nanoprobes, especially in Surface-Enhanced Raman Spectroscopy (SERS) or colorimetric assays, offer rapid and label-free signal generation, making them suitable for field-deployable or bedside settings. However, their optical response can be affected by sample complexity and ambient conditions, reducing reliability in whole-blood matrices [54].

Zhou et al. [55], by contrast, emphasized smart magnetic probes and microfluidic-integrated sensors with enhanced sensitivity and real-time imaging capabilities. This approach, while scientifically robust, often relies on high-end instrumentation, limiting its accessibility and scalability in typical clinical environments. This comparison underscores a key trade-off between simplicity and operational power, especially when considering resource-constrained settings. From a performance standpoint, Pant et al. [56] highlighted electrochemical biosensors employing zinc oxide (ZnO) nanotubes, carbon nanofibers, and AuNPs for CRP and PCT detection. These systems demonstrated ultralow detection limits (as low as 0.05 ng mL^−1^), superior specificity, and compatibility with portable formats. Nevertheless, their technical demands, such as multi-step probe immobilization and surface functionalization, pose challenges in mass production and long-term stability. Vasconcelos et al. [57] contributed by showcasing quantum dot- and immunomagnetic-based platforms capable of multiplexing multiple sepsis biomarkers using a very small sample volume. While this multiplex capability is advantageous in critical care, particularly in neonates or patients with a limited blood volume, many of these technologies remain in preclinical phases, constrained by biocompatibility concerns, regulatory hurdles, and high production costs.

These examples highlight the strong potential of nanotechnology-based biosensors but also underline the key challenges that must be addressed before they can be widely used in clinical settings. Although promising for bedside or point-of-care diagnostics, several issues remain, such as signal interference in complex samples, the lack of clinical validation, and inconsistent performance across different patients. In addition, practical barriers, like high production costs, limited scalability, and safety concerns, around nanomaterials (e.g., graphene and quantum dots) still need solutions. Moving forward, the goal is to develop biosensors that are not only sensitive and specific but also affordable, reliable, and easy to use in real-world healthcare settings. Combining the portability of electrochemical sensors with the multiplexing ability of optical and magnetic platforms may offer a balanced approach. To be truly impactful, these technologies must also be accessible in low- and middle-income countries, where the burden of sepsis is often highest. Continued innovation in this area is crucial for improving early detection and saving lives.

#### 2.2.5. Commercial Sepsis Diagnosis Kits

Commercial sepsis detection kits provide rapid and accurate pathogen identification for the speedy diagnosis and treatment of sepsis illnesses. These kits utilize technologies like PCR, lateral flow, BCs, Enzyme-Linked Immunosorbent Assay (ELISA), and immunochromatographic assays to detect sepsis-causing pathogens and biomarkers (Table 1) [58,59,60,61,62,63,64,65,66,67,68,69]. They offer advantages over traditional culture methods by providing results in a shorter timeframe, and they monitor signs of worsening sepsis, which is crucial in the intensive care setting. Most sepsis rapid kits focus on detecting a single biomarker, and while some kits may detect multiple biomarkers, the majority are designed for single-target detection. While sepsis diagnostic kits, like those using rapid detection techniques, can be valuable tools, they still require competent personnel for proper handling and use, alongside an accurate interpretation of the results. For instance, the sepsis diagnosis test kit based on PCR assay involves necessary reagents for the amplification of specific DNA or RNA sequences. Fluorescent probes or dyes are incorporated, in which their signals are measured for each partition individually to determine the presence and quantity of the target molecules.

## 3. Electrochemical and Aptamer-Based Platforms: The Frontier of Sepsis Diagnostics

### 3.1. Principles of Electrochemical Biosensor Operation

Electrochemical biosensors are highly relevant for sepsis detection, as they translate biological recognition events, such as the binding of biomarkers, e.g., PCT or CRP, into measurable electrochemical signals, which relate to the target molecule concentration levels at the transducer surface. These biosensors rely on electrochemical transducers, like voltammetry, potentiometry, amperometry, and impedimetry, to quantify biomarker levels in patient samples, offering rapid and sensitive diagnostics. In sepsis detection, electrochemical sensors are modified with nanomaterials (e.g., AuNPs, carbon nanotubes (CNTs), graphene oxide (GO), magnetic Fe_3_O_4_@Au, etc.) to enhance the sensitivity by improving the electron transfer and increasing the active surface area. The recognition element, such as the immobilized aptamers or antibodies, specifically binds to the sepsis biomarker, leading to changes in the current, voltage, or impedance signal, which is then analyzed through electrochemical techniques, e.g., cyclic voltammetry (CV), differential pulse voltammetry (DPV), chronoamperometry, potentiometry, and impedimetry.

**Figure 2:**
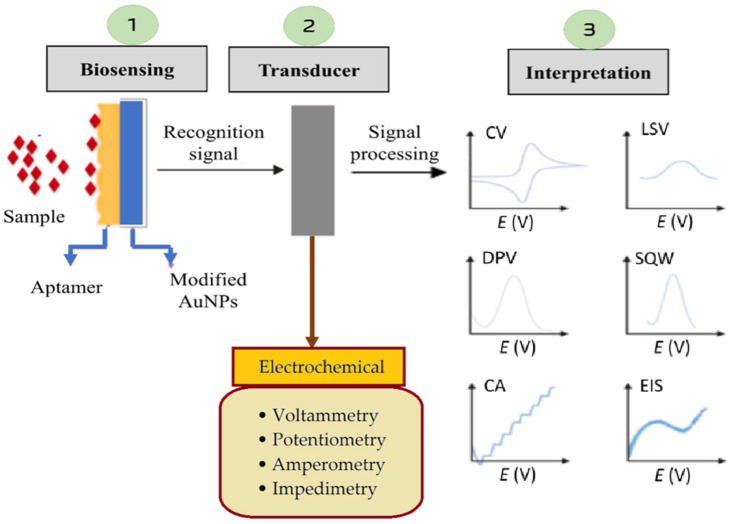
Electrochemical signal processing in biosensor-based sepsis detection. The biosensing step (1) involves target recognition through aptamer–analyte binding on modified gold nanoparticles (AuNPs), generating a recognition signal. The transducer unit (2) converts this biological interaction into an electrochemical signal, which is then processed and interpreted (3) using various techniques, such as cyclic voltammetry (CV), differential pulse voltammetry (DPV), chronoamperometry (CA), linear sweep voltammetry (LSV), square wave voltammetry (SWV), and electrochemical impedance spectroscopy (EIS).

The potentiometric transducer converts the changes in the distribution of charges into an electrical signal [70]. The signal is typically measured in the form of a current at a given potential, which allows for precise quantification of the target analyte. The movement of electrons produced in a redox reaction, on the other hand, can be measured by an amperometric transducer in the form of an electrical current signal [71]. A voltammetric transducer measures a current as a function of a controlled electrode potential and time, which results in a current–voltage display [72,73,74], and an impedance transducer registers changes in electrical properties either in the form of capacitance or resistance at the electrode–electrolyte interface as a result of interactions between the biorecognition element attached to the transducer surface and the analyte present in a sample solution [75]. Figure 2 illustrates a detailed representation of electrochemical signal processing in biosensor-based diagnostics. Compared to conventional methods, like ELISA or PCR, electrochemical biosensors offer real-time, cost-effective, and portable solutions for early sepsis diagnosis, crucial for timely clinical intervention.

### 3.2. Significance of Aptamers in Sepsis Biosensing as Superior Alternatives to Antibodies

Aptamers have emerged as powerful molecular recognition elements in biosensor technology, offering critical advantages over conventional antibody-based systems, particularly in the context of sepsis diagnostics [76]. Unlike antibodies, which require complex in vivo production, aptamers are generated via the Systematic Evolution of Ligands by Exponential Enrichment (SELEX) process, allowing for fully in vitro synthesis with high batch reproducibility, tunable specificity, and low costs [77]. Chemically stable and thermally resilient aptamers can be modified with diverse functional groups and integrated into a wide range of detection platforms, e.g., electrochemical, optical, colorimetric, and magnetic ones, without compromising their structural integrity [17,78]. This modularity is particularly advantageous for POC sepsis diagnostics, where speed, miniaturization, and multiplexing are essential.

Several studies have demonstrated that aptamer-based biosensors match or exceed antibody-based systems in sensitivity, often reaching femtomolar or even attomolar detection limits for sepsis-related biomarkers, such as IL-6, CRP, and tumor necrosis factor-alpha (TNF-α) [17,79]. Notably, by creating universal aptamers (i.e., Antibac1 and Antibac2) against bacterial peptidoglycan, Graziani et al. [80] accomplished a remarkable achievement that is rarely possible with a single antibody, robust binding across a wide range of gram-positive and gram-negative sepsis pathogens. Similarly, aptamers targeting whole bacterial cells, such as *S. aureus*, *E. coli*, and *K. pneumoniae*, have demonstrated exceptional specificity and nanomolar-to-picomolar dissociation constants, outperforming immunoassays in both breadth and turnaround time [81,82]. Moreover, aptamer–nanomaterial hybrids have shown synergistic enhancements in signal transduction and target accessibility, enabling low-cost, label-free biosensors with superior detection dynamics [83].

Beyond their physicochemical superiority, aptamers also address important translational and logistical bottlenecks in diagnostic deployment, particularly in resource-limited or time-critical settings, such as emergency care and intensive care units [84]. The in vitro synthesis of aptamers circumvents the ethical, cost, and variability issues associated with animal-based antibody production and facilitates rapid re-engineering to adapt to newly emerging pathogens or biomarker variants. This was exemplified in the work by Saad and Faucher [81], where aptamers selected via cell-SELEX maintained binding integrity across environmental conditions, enabling stable detection of diverse water- and environment-associated pathogens relevant to healthcare-acquired infections [81]. Similarly, the dual aptamer constructs developed by Graziani et al. [80] were shown to bind conserved bacterial cell wall structures across eleven bacterial species, highlighting the potential of aptamers to serve as pan-bacterial probes for early-stage bloodstream-infection screening. These capabilities contrast sharply with antibodies, which often require antigenic epitope preservation, are less tolerant of storage stress, and typically necessitate separate optimization for each target.

Moreover, aptamers enable the integration of next-generation biosensing functions, such as real-time monitoring, miniaturization, and wireless signal transmission. As demonstrated by Liu et al. [17], aptamer-based platforms interfaced with nanostructured materials, such as AuNPs and GO, achieved exceptional sensitivity for both pathogen detection (as low as 100 CFU mL^−1^) and cytokine quantification in complex matrices [17]. Their review also emphasized the potential of aptamers to function in homogeneous, wash-free assays, drastically reducing processing times. Further supporting this, Sequeira-Antunes and Ferreira [78] and Li et al. [79] illustrated that aptamers could be functionalized for electrochemical signal generation, allowing for label-free quantification and multiplex analysis on compact lab-on-chip devices. In clinical contexts like sepsis, each hour of diagnostic delay increases mortality risk by 7.6%. The advancement in electrochemical aptasensors’ performance features represents not just technical enhancements but lifesaving improvements. Thus, aptamers not only represent an innovative alternative to antibodies, but they also serve as a bridge to a new era of intelligent, decentralized, and high-throughput diagnostic systems.

### 3.3. Recent Electrochemical Aptamer-Based Biosensors for Sepsis Biomarkers (January 2020–May 2025)

Over the past five years, electrochemical aptamer-based biosensors have emerged as a central innovation in the diagnostic landscape for sepsis. Aptamers, synthetic oligonucleotides selected for high affinity and specificity, offer clear advantages over antibodies, including chemical stability, cost-effective synthesis, tunable binding dynamics, and easier integration into electronic interfaces. These properties are especially attractive for developing biosensors that can meet the World Health Organization’s ASSURED criteria (i.e., Affordable, Sensitive, Specific, User-friendly, Rapid, Equipment-free, and Deliverable) for POC diagnostics [85]. Electrochemical platforms, with their miniaturization potential, fast response, and amenability to label-free detection, are ideal transduction systems for aptamer integration. Between 2020 and 2025, a surge of research has focused on designing electrochemical aptasensors capable of detecting sepsis biomarkers with high sensitivity and rapid turnaround. These biosensors typically target one or more of the following: acute-phase reactants, such as C-reactive protein (CRP); cytokines, like interleukin-6 (IL-6) and tumor necrosis factor-alpha (TNF-α); procalcitonin (PCT); microRNAs; and bacterial endotoxins, like lipopolysaccharides (LPSs). Table 2 provides a comparative summary of 13 recent aptasensor platforms, highlighting differences in design, transduction strategies, performance metrics, and diagnostic applicability.

A pioneering study by Firoozbakhtian et al. [86] introduced a carbon nanotube (CNT)-based field-effect transistor (FET) aptasensor for CRP, leveraging a buried-gate architecture to achieve a rapid response (8 min) and a detection limit of 150 pM, with high specificity against inflammatory cytokines, like IL-6 and TNF-α. This system demonstrated strong potential for label-free, real-time detection in a compact format. In parallel, Whitehouse et al. [87] developed a structure-switching electrochemical aptasensor (SS-EABs) that enabled reagentless, single-step CRP detection within 1 min in serum. Its reusability and week-long stability offered practical advantages over conventional ELISA assays, especially for decentralized and repeated patient monitoring.

Expanding beyond CRP, IL-6 detection has become a critical focus due to its close correlation with inflammation severity and disease progression. Diacci et al. [88] designed an organic electrochemical transistor (OECT) platform functionalized with AuNPs and thiolated aptamers, allowing for IL-6 quantification down to picomolar levels with good selectivity against TNF. Sánchez-Salcedo et al. [89] presented a capacitive electrochemical impedance spectroscopy (EIS) aptasensor for IL-6 that performed comparably to nanobody-based counterparts in human serum, while offering superior cost-efficiency, stability, and potential for wearable integration. Notably, Ondevilla et al. [90] demonstrated a rapid, multiplex aptamer-based biosensor capable of detecting IL-6, TNF-α, and miRNA-155 in LPS-induced septic mice, achieving femtogram-level sensitivity with electrokinetic-enhanced microelectrodes. This platform significantly reduced the detection time to under 5 min, positioning it as a potential alternative to standard immunoassays.

In efforts to overcome the limitations of single-analyte biosensors, hybrid strategies have emerged. Yang et al. [91] introduced a dual-recognition platform combining aptamers and antibodies, conjugated onto AuNPs, enabling PCT detection from 10 ng mL^−1^ to 100 ng mL^−1^ with a strong linear correlation (R^2^ = 0.9937) in serum. This aptamer–antibody synergy improved both selectivity and signal output, highlighting the benefits of biomolecular complementation. Meanwhile, Alvandi et al. [92] developed an MXene-GO composite-based FET biosensor to detect both *Escherichia coli* (*E. coli*) and its LPS endotoxin. Aptamer-functionalized surfaces enabled rapid (300 s) and sensitive (1 pg mL^−1^ LPS) detection even in untreated human blood, demonstrating excellent real-world applicability and 30-day stability.

Lipopolysaccharides (LPSs), a key component of the outer membrane of gram-negative bacteria, serve as an early biomarker for sepsis caused by these pathogens. Their clinical relevance has driven the development of diverse biosensor strategies for timely detection. Mobed et al.’s [93] review of LPS biosensors emphasized how aptamers, when integrated with electrochemical platforms and nanomaterials, enhance sensitivity into the femtomolar range. Zhu et al. [94] extended this by introducing a nanopore aptasensor for single-molecule LPS identification, capable of distinguishing serotypes in untreated serum at 10 ng mL^−1^, underscoring its potential in precision diagnostics. To improve detection sensitivity, Wang et al. [95] employed phenylboronic acid (PBA)-modified graphene to interact with LPSs’ cis-diol structures, achieving a detection limit as low as 3.9 fg mL^−1^. Yu et al. [96] advanced this further by integrating photo-initiated polymerization with ferrocene tagging, allowing for femtogram-level LPS detection (limit of detection (LOD) = 0.25 fg mL^−1^) via a dual-amplification electrochemical strategy.

**Table 2:** Performance comparison of electrochemical aptamer-based biosensors for sepsis diagnosis (2020–2025).

Sensor Type	Target Biomarker	LOD	Linear Range	Response Time	Non- Clinical Matrix	Clinical Matrix	Electrochemical Transducer	Notable Features (Strengths)	Limitations	Reference
CNT-FET aptasensor	CRP	150.000 pM	0.05 – 5.00 mg L^−1^	8 min	Buffer	-	FET	Real-time label-free detection and high stability	Tested only in buffer; CRP overlaps with non-septic inflammation	[86]
Structure-switching EAB	CRP	20.000 – 60.000 nM	1.00–500.00 nM	1 min		50% human serum	Voltammetry	Single-step detection, reagentless, and reusable	Limited-to -moderate CRP levels; long-term stability untested	[87]
OECT aptasensor	IL-6	60.000 pM	pM–nM	-	Buffer	Human serum	OECT (conductance modulation)	Low-voltage, aqueous compatibility, and miniaturizable	Selective for IL-6 but lacks multiplex capability	[88]
Capacitive EIS aptasensor	IL-6	5.000 pg mL^−1^	5.00 pg mL^−1^ – 1.00 ng mL^−1^	-	-	10% human serum	EIS	Capacitive mode, flexible sensor, and suitable for wearables	Capacitive EIS is less established in POC applications	[89]
Multiplex aptasensor with electrokinetic strip	IL-6, TNF-α, and miRNA-155	0.180 pg mL^−1^ (IL-6), 0.840 pg mL^−1^ (TNF-α), 0.001 pg mL^−1^ (miRNA-155)	-	5 min	-	Murine sepsis serum	Amperometry/voltammetry (multiplex)	Multiplex detection, femtogram sensitivity, and fast hybridization	Needs validation in human serum; animal models only	[90]
IDE AuNP-aptamer– antibody	PCT	10.000 ng mL^−1^	10.00 – 100.00 ng mL^−1^	-	Spiked serum	-	Impedance/ amperometric (IDE)	Hybrid probe (aptamer + antibody) and good correlation in serum	Moderate sensitivity; aptamer/antibody ratio optimization needed	[91]
MXene-GO FET aptasensor	LPSs and *E. coli*	1.000 pg mL^−1^ (LPS), 3.000 CFU mL^−1^ (*E. coli*)	-	5 min	-	Human serum	FET	Rapid whole-cell and LPS detection, stable, and portable	Fabrication complexity; long-term reproducibility not shown	[92]
Nanopore aptasensor	LPSs	10.000 ng mL^−1^	-	Fast	Tap water	Human serum	Nanopore current blockade	Single-molecule detection and serotype resolution	Needs specialized nanopore equipment	[94]
Graphene-PBA aptasensor	LPSs	3.900 fg mL^−1^	-	-	-	-	Amperometry (graphene interface)	Signal amplification via PBA-cis-diol binding	Matrix interference not fully explored	[95]
Photo-ATRP amplified aptasensor	LPSs	0.250 fg mL^−1^	1.00 fg mL^−1^ – 0.10 pg mL^−1^	<4.5 h	-	Human serum	Photo-ATRP with ferrocene polymerization	Dual amplification (chemical + photocatalytic) and ultralow LOD	Longer detection time; red-light setup required	[96]
Magnetic Fe_3_O_4_@Au aptasensor	HSP70	0.525 pg mL^−1^	10.00 pg/mL – 200.00 ng/mL	-	-	Human serum	Impedance + magnetic enrichment	Magnetic recovery, reusability, and good performance in blood	Sensor regeneration steps may reduce throughput	[97]

**Figure 3:**
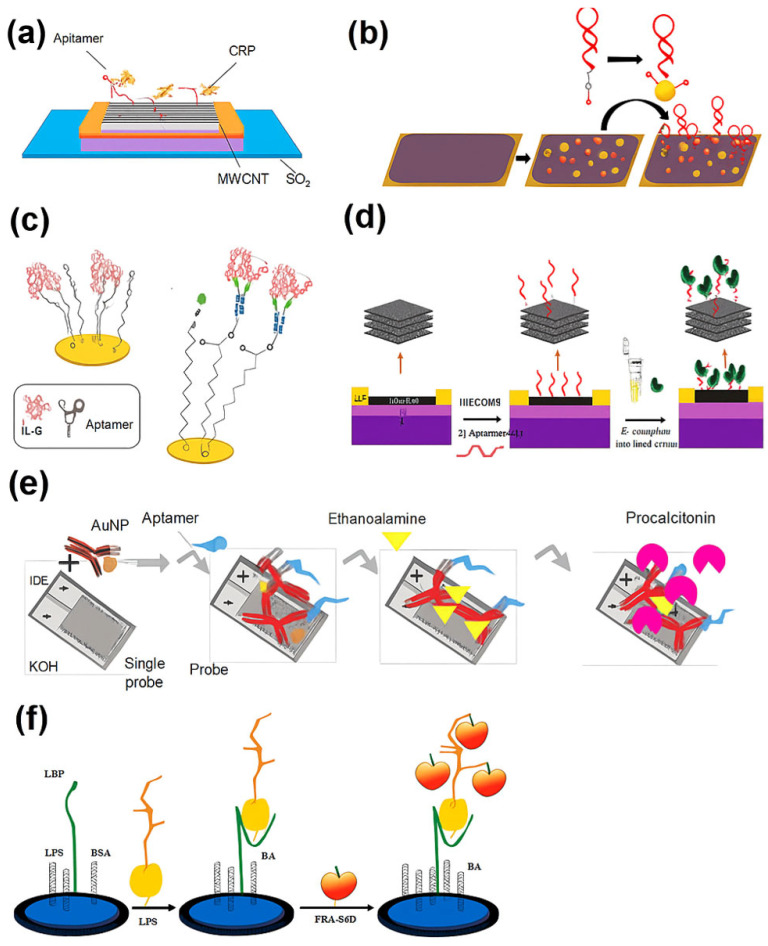
Schematic illustrations of electrochemical aptamer-based biosensors for sepsis biomarker detection. (**a**) CNT-FET aptasensor for CRP detection with a buried-gate transistor; (**b**) OECT aptasensor functionalized with AuNPs for IL-6 quantification; (**c**) EIS aptasensor platform in human serum for IL-6; (**d**) MXene-GO composite FET aptasensor for *E. coli* and LPS detection; (**e**) dual-recognition aptasensor combining aptamers and antibodies on AuNPs for PCT detection; (**f**) PBA-graphene electrochemical platform for LPS cis-diol binding.

Beyond traditional markers, Liu et al. [97] introduced a reusable magnetic electrochemical aptasensor targeting heat shock protein 70 (HSP70), a stress-response protein associated with septic inflammation. This biosensor employed Apt7-functionalized Au-coated Fe_3_O_4_ (Fe_3_O_4_@Au) nanoparticles magnetically immobilized onto a working electrode, achieving a detection range from 10 pg mL^−1^ to 200 ng mL^−1^ with superior accuracy in serum compared to ELISA [97]. Figure 3 illustrates selected examples of electrochemical aptamer-based biosensor designs from recent studies, highlighting differences in transduction methods, recognition strategies, and biomarker targets relevant to sepsis diagnostics. These examples highlight key stages in biosensor operation, including aptamer immobilization, target binding and conformational switching, and electrochemical signal transduction. This figure also shows diverse design strategies from recent studies, demonstrating how innovations in material interfaces and signal amplification contribute to improved sensitivity, selectivity, and point-of-care applicability.

While these biosensors exhibit compelling performance in terms of speed, sensitivity, and specificity, some challenges remain. Multiplexing capabilities are not yet universally integrated, and long-term reproducibility and real-world clinical trials are often lacking. Some systems also rely on complex fabrication or specialized materials, e.g., MXene or red-light-driven catalysis, which may limit scalability. Nevertheless, the continuous evolution of aptamer engineering, surface chemistry, and transduction technology strongly suggests that these biosensors will play a central role in the future of sepsis diagnostics, particularly in resource-limited and point-of-care settings. To contextualize this trajectory, it is essential to visually synthesize how these platforms align with current biomarker targets, diagnostic capabilities, and clinical translation pathways. The current technologies concerning their advantages and disadvantages with respect to electrochemical aptasensors for sepsis diagnosis are summarized in Table 3.

**Figure 4:**
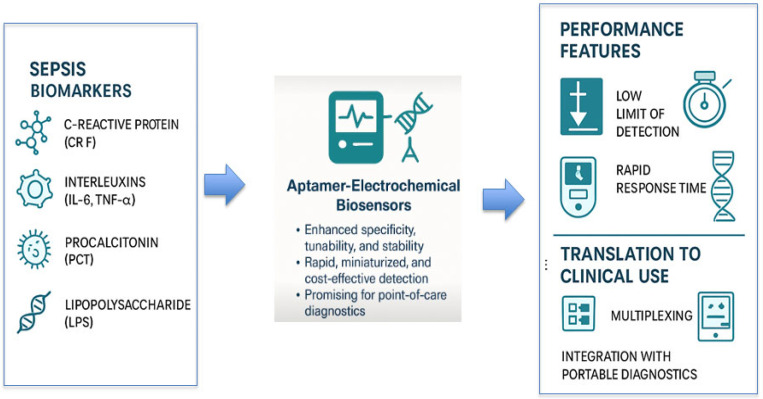
Overview of aptamer–electrochemical biosensors for sepsis diagnostics. This schematic highlights key target biomarkers, including CRP, interleukins (IL-6 and TNF-α), PCT, and LPSs, alongside the unique performance advantages of aptamer-integrated electrochemical platforms, such as low detection limits and rapid response. These attributes support their potential for translation into clinical applications, particularly through multiplexing capabilities and integration with portable diagnostic tools.

The integration of aptamers with electrochemical transduction systems represents a pivotal advancement in biosensing strategies for sepsis. As illustrated in Figure 4, these platforms are engineered to detect a range of clinically relevant biomarkers, including CRP, IL-6, TNF-α, PCT, and LPSs with enhanced specificity, tunability, and speed. Their ability to achieve low detection limits in compact, miniaturized formats supports rapid diagnostics at the point of care. More importantly, the evolution of these biosensors toward multiplex detection and compatibility with mobile and wearable diagnostics underscores their growing translational potential. This schematic captures the essential components and functional strengths of electrochemical aptasensors, serving as a visual summary of their trajectory from conceptual design to clinical readiness.

## 4. Critical Assessment and Future Outlook

Despite the impressive strides made in electrochemical aptamer-based biosensing for sepsis biomarkers between 2020 and 2025, several limitations remain that hinder widespread clinical adoption. Most notably, the translational gap between proof-of-concept and real-world applications is yet to be bridged. While demonstrating ultralow detection limits and rapid response times, a significant portion of the reported biosensors have only been validated in buffered solutions or spiked serum. Clinical matrices, such as whole blood, plasma from septic patients, or polymicrobial samples, present far greater complexity, including biofouling, nonspecific adsorption, and variable analyte levels that fluctuate with disease progression. Furthermore, although many platforms exhibit picomolar to femtomolar sensitivity, sensitivity alone does not guarantee clinical utility. For example, while LODs in the femtogram range may seem attractive, they often require specialized materials, elaborate surface chemistry, or long signal amplification steps that are incompatible with POC use. Simpler systems, even if slightly less sensitive, may be more valuable if they can deliver reliable and reproducible results within 15–30 min using finger-prick volumes of blood and minimal processing.

Another major challenge is standardization. There is a lack of consensus on performance metrics, test conditions, and validation protocols. Comparisons across studies are often confounded by differing matrices, concentrations, or definitions of LOD. This variability underscores the urgent need for standardized benchmarking frameworks, perhaps modeled after the Minimum Information for Publication of Quantitative Real-Time PCR Experiments (MIQEs) guidelines established for molecular diagnostics. Such a framework should mandate the comprehensive reporting of critical biosensor validation parameters, including (i) dynamic range, defined by both lower and upper limits of quantification; (ii) reproducibility, characterized by intra- and inter-assay variation; (iii) matrix compatibility, with clear differentiation between buffer-based, spiked, and clinical samples; (iv) response time and signal decay profiles under realistic operating conditions; (v) selectivity assessments against non-target analytes; and (vi) calibration procedures and statistical methods used for data interpretation. By adopting unified criteria for biosensor evaluation, researchers can ensure greater transparency, facilitate cross-platform comparisons, and support regulatory appraisal, thereby accelerating the deployment of biosensor technologies into routine clinical workflows for sepsis diagnosis.

Beyond analytical validation, successful clinical translation also requires a clear pathway through regulatory and clinical trial frameworks. Biosensors intended for diagnostic use must undergo staged validation processes, beginning with analytical verification of performance metrics, such as sensitivity, specificity, and reproducibility, under controlled laboratory conditions. This is typically followed by clinical performance studies in real-world patient samples, ideally across multiple institutions, to assess diagnostic accuracy against current gold standards. Regulatory clearance varies by region: in the United States, biosensors are often classified as in vitro diagnostic (IVD) devices and assessed under the FDA’s 510(k) or Premarket Approval (PMA) pathways, depending on the risk level [98]. In the European Union, the In Vitro Diagnostic Regulation (IVDR) framework outlines conformity assessment and risk classification for diagnostic devices. Additionally, point-of-care biosensors may require compliance with CLIA (Clinical Laboratory Improvement Amendments) to ensure usability outside centralized labs. Early integration of regulatory expectations into biosensor design can streamline approval, reduce delays, and support alignment with clinical workflows.

In addition, the field also faces a critical trade-off between multiplexing and operational complexity. While several platforms target individual biomarkers with high sensitivity, sepsis is inherently a multi-factorial condition. Diagnosis and staging require the simultaneous profiling of inflammatory markers (IL-6 and TNF-α), bacterial components (LPSs), stress proteins (HSP70), and host–response indicators (PCT, CRP, and miRNAs). However, many current aptasensors are not designed to handle multiplexed detection, or they require complex signal deconvolution schemes. The few systems that do achieve multiplexing often face limitations in fabrication scalability, cross-reactivity, or increased assay times.

On the materials front, while innovations like MXene, boronic acid-functionalized graphene, and photo-initiated polymerization have shown substantial promise, questions of cost, environmental stability, and regulatory acceptability remain. MXenes, for instance, have shown exceptional conductivity and surface functionality, but concerns over oxidation, biocompatibility, and reproducibility in manufacturing must be resolved. Likewise, light-activated or chemically amplified sensors may be less viable for field deployment unless simplified and miniaturized. Looking forward, future progress will require a system-level approach. This includes integrating aptasensors with microfluidic sample handling, real-time signal processing, smartphone-based readout systems, and cloud-connected databases for epidemiological tracking. Advances in machine learning can further accelerate aptamer selection, enabling in silico evolution of high-affinity sequences tailored to complex targets or multiplex panels. The development of reusable sensor cartridges with self-cleaning or anti-fouling surfaces could also reduce costs and improve sustainability, particularly in low-resource settings.

Most importantly, collaborations between materials scientists, clinical microbiologists, and bioengineers must intensify. Clinical validation needs to be at the forefront of biosensor development, not an afterthought. Future studies should prioritize patient-derived samples, multi-center trials, and regulatory readiness. Bridging the lab-to-clinic divide will not only require innovation but also alignment with healthcare workflows, data interoperability, and human-centered design. Hence, electrochemical aptamer-based biosensors are no longer an emerging technology. They are on the cusp of clinical relevance. However, realizing their full potential in sepsis diagnostics will depend on a new generation of biosensors that are not just more sensitive but smarter, simpler, and truly integrated with the realities of clinical care, grounded in clinical validation, and aligned with regulatory readiness.

## 5. Conclusions

This review critically assessed the role of aptamer-based electrochemical biosensors in advancing sepsis diagnostics. The integration of aptamers offers distinct advantages, including high specificity, chemical stability, and the ability to target a wide range of sepsis biomarkers. Coupled with electrochemical transduction, these biosensors present a compelling approach for developing rapid, low-cost, and miniaturized diagnostic platforms suitable for point-of-care (POC) applications. Despite promising performance in terms of sensitivity and detection speed, most reported systems remain at the proof-of-concept stage, with limited validation in clinically relevant matrices. Challenges such as standardization of evaluation protocols, reproducibility across platforms, and regulatory considerations continue to hinder clinical translation. Moreover, many systems focus primarily on analytical performance, often overlooking factors essential for real-world implementation, such as usability, integration with clinical workflows, and patient diversity. To advance this field, future research should emphasize translational studies involving patient-derived samples, real-world benchmarking, and interdisciplinary collaboration. Addressing these gaps will be essential to unlock the full clinical utility of aptamer-based biosensors in sepsis management and beyond.

## Figures and Tables

**Table 1:** Commercial sepsis detection kits based on PCR, lateral flow, blood culture, ELISA, and immunochromatographic assays to detect sepsis-causing pathogens and biomarkers.

Method	Product	Target Biomarker	LOD	Response Time	Matrix	Quantification Range	Qualitative/Quantitative	Manufacturer	Reference
Digital PCR	Sepsis Pathogenic Microorganism Detection Kit	21 bacteria and fungi	3–5 copies µL^−1^	<5 h	Whole blood	-	Both	RainSure SCIENTIFIC	[58]
Lateral flow	Rapid sepsis test SeptiLoop	Bacteria	-	3 h	Whole blood	-	Quantitative	Loop Diagnostics	[59]
	PCT test kit	PCT	-	15 min	Serum and plasma	-	Quantitative	Guangzhou KOFA Biotechnology Co., Ltd.	[60]
	Sepsis test kit Optical Q™	PCT	-	15 min	Blood, serum, and tissue	0.10–100.00 ng mL^−1^	Quantitative	OptiBio Co., Ltd.	[61]
	Sepsis test kit	PCT	-	12 min	Serum, plasma, and whole blood	0.1–100 ng mL^−1^	Quantitative	Boditech Med Inc.	[62]
Blood cultures	Sepsis test kit BCID	Virus, bacteria, fungi	-	1.5 h	Whole blood	-	Quantitative	GenMark Diagnostics	[63]
ELISA	Sepsis assay kit PATHFAST™	PCT	-	17 min	Serum, plasma, and whole blood	-	Quantitative	PHC Europe B.V./PHCbi	[64]
	PCT assay kit Lumipulse^®^ G	CRP, PCT	-	6 h	Serum and plasma	-	Quantitative	Fujirebio	[65]
Fluorescence immunochromatography	Sepsis test kit OTK002	PCT	-	-	Serum, plasma, and whole blood	-	Quantitative	Hangzhou Singclean Medical Products	[66]
	Sepsis test kit OTK004	IL-6	-	-	Serum, plasma, whole blood, and bone marrow	-	Quantitative	Hangzhou Singclean Medical Products	[67]
	PCT test kit	PCT	-	15 min	Serum and plasma	0.02–400 ng mL^−1^	Quantitative	Nanjing Vazyme Medical Technology Co., Ltd	[68]
Fluorescence immune assay	Sepsis test kit FiCA™	PCT	0.1–100 ng mL^−1^	-	Serum, plasma, and whole blood	-	Quantitative	Medlere Limited	[69]
	Sepsis test kit IF 1088	IL-6	-	15 min	Serum, plasma, and whole blood	-	Quantitative	Getein Biotech Inc.	[69]

**Table 3:** Summary of current technologies concerning their advantages and disadvantages for sepsis diagnosis.

Sepsis Detection Technologies	Advantages	Disadvantages
BCs	Gold standard for detecting bloodstream infections. Low-cost and accessible procedure. Essential for antibiotic susceptibility testing.	Requires large blood volumes. Slow nature of the blood culture procedure. Requires a 24–72 h diagnostic turnaround time. Potential false-positive or -negative detection. Limited sensitivity when pathogen load is low. Patients may miss out on important medical interventions. Difficulty in detecting fungal and viral causes of sepsis.
PCR-based techniques	Allows for pathogen detection in a few hours by amplifying specific nucleic acid sequences. Holds promise for improving sensitivity upon integration with NGS molecular techniques.	Less accessible in resource-limited settings. Reliance on primer specificity prevents the detection of all bacterial strains. Sample contamination leads to false positives or incorrect results. Low pathogen concentration complicates the diagnostic process. Does not provide information on antimicrobial resistance. High costs and complex PCR-based techniques. Requires specialized equipment and expertise. Sensitivity may be compromised by the presence of host DNA or interferents in the blood samples.
ELISA	Qualitative and quantitative analyses are available. Offers high sensitivity and high-throughput screening.	Require complex in vivo production using living organisms. Antibodies utilized as recognition elements are highly sensitive to various physical and chemical conditions. Requires antigenic epitope preservation and separate optimization for each target. Requires long incubation hours (>2–3 h). High potential for false-positive/negative results.
Spectroscopy-based approaches	High sensitivity. High-throughput capabilities. Rapid identification of pathogens in sepsis. Enable the simultaneous identification of a wide variety of pathogens. An essential complement to conventional BC technique.	High costs and requires highly trained personnel. Less accessible in under-resourced settings. Potential false-positive or -negative detection due to the contingency on pathogen culture. Unable to detect antimicrobial resistance. Requires well-maintained microbial databases to ensure the accuracy of pathogen identification.
Nanotechnology-based sensors/biosensors	Offers improved sensitivity, specificity, and real-time detection. Offers rapid and label-free signal generation. Potential application in portable POC diagnostics. Requires a very small sample volume. Capable of multiplexing multiple sepsis biomarkers.	Optical transduction can be affected by sample complexity (e.g., whole blood) and ambient conditions. Nanomaterial-based electrodes require multi-step probe immobilization and surface functionalization, posing challenges in mass production and long-term stability. Microfluidic-integrated sensors with limited accessibility and scalability in typical clinical environments. Technologies remain in preclinical phases, constrained by biocompatibility concerns, regulatory hurdles, and high production costs. Clinical validation remains a crucial barrier.
Electrochemical aptasensors	In vitro synthesis of aptamers circumvents ethical, cost, and variability issues associated with animal-based antibody production. Chemically stable and thermally resilient. Aptamers can be modified with diverse functional groups for integration into electronic interfaces. Offers rapid, high-throughput, label-free, multiplexing, sensitive, cost-effective, and POC sepsis diagnostics. Aptamer–nanomaterial hybrids render signal transduction enhancement and target accessibility. Tunable binding dynamics and facilitate rapid re-engineering to adapt to newly emerging pathogens or biomarker variants. Enables integration of next-generation biosensing functions, i.e., real-time monitoring, miniaturization, and wireless signal transmission. Offers homogeneous and wash-free assays.	Surface modification and signal amplification strategies are essential.

## Data Availability

No new data were created or analyzed in this study. Data sharing is not applicable to this article.

## Abbreviations

The following abbreviations are used in this manuscript.

ACCP	American College of Chest Physicians
ASSURED	Affordable, Sensitive, Specific, User-friendly, Rapid, Equipment-free, and Deliverable
AuNPs	Gold nanoparticles
BCs	Blood cultures
CA	Chronoamperometry
CNTs	Carbon nanotubes
CRP	C-reactive protein
CV	Cyclic voltammetry
DPV	Differential pulse voltammetry
EIS	Electrochemical impedance spectroscopy
ELISA	Enzyme-Linked Immunosorbent Assay
FET	Field-effect transistor
Fe_3_O_4_@Aus	Au-coated Fe_3_O_4_ nanoparticles
HSP70	Heat shock protein 70
IDE	Interdigitated electrode
ILs	Interleukins
IL-6	Interleukin-6
LMICs	Low- and middle-income countries
LOD	Limit of detection
LPSs	Lipopolysaccharides
LSPR	Localized surface plasmon resonance
LSV	Linear sweep voltammetry
MALDI-TOF	Matrix-Assisted Laser Desorption/Ionization Time of Flight
MIQEs	Minimum Information for Publication of Quantitative Real-Time PCR Experiments
MM	Micromotor
MODS	Multiple organ dysfunction syndrome
MXene-GO FET	Field-effect transistor aptasensor based on MXene and graphene oxide
NGS	Next-generation sequencing
OECT	Organic electrochemical transistor
PBA	Phenylboronic acid
PCR	Polymerase chain reaction
PCT	Procalcitonin
POC	Point of care
SCCM	Society of Critical Care Medicine
SELEX	Systematic Evolution of Ligands by Exponential Enrichment
SERS	Surface-Enhanced Raman Spectroscopy
SIRS	Systemic inflammatory response syndrome
SPR	Surface plasmon resonance
SS-EABs	Structure-switching electrochemical aptamer-based sensors
SWV	Square wave voltammetry
TNF-α	Tumor necrosis factor-alpha
